# The burden of serious non-AIDS-defining events among admitted cART-naive AIDS patients in China: An observational cohort study

**DOI:** 10.1371/journal.pone.0243773

**Published:** 2020-12-22

**Authors:** Yu Wang, Hongyuan Liang, Ling Zhang, Zhe Zhang, Liang Wu, Liang Ni, Guiju Gao, Di Yang, Hongxin Zhao, Jiang Xiao

**Affiliations:** Clinical and Research Center of Infectious Diseases, Beijing Ditan Hospital, Capital Medical University, Beijing, China; Centers for Disease Control and Prevention, UNITED STATES

## Abstract

The objective of this study was to elucidate the burden, risk factors, and prognosis of serious non-AIDS-defining events among admitted cART-naive AIDS patients in China. The evaluation of the burden, risk factors and prognosis of serious NADEs was carried out among 1309 cART-naive AIDS patients (median age: 38.2 years, range: 18–78 years) admitted in Beijing Ditan Hospital between January 2009 and December 2018. Among 1309 patients, 143 patients (10.9%) had at least one serious NADEs, including 49 (3.8%) with cerebrovascular diseases, 37 (2.8%) with non-AIDS-defining cancers, 28 (2.1%) with chronic kidney diseases, 26 (2.0%) with cardiovascular diseases, and 18 (1.4%) with liver cirrhosis. Serious NADEs distributed in different age and CD4 levels, especially with age ≥50 years and CD4 ≤350 cells/ul. Other traditional risk factors, including cigarette smoking (OR = 1.9, 95%CI = 1.3–2.8, p = 0.002), hypertension (OR = 2.5, 95%CI = 1.7–3.7, p<0.001), chronic HCV infection (OR = 2.8, 95%CI = 1.4–5.6, p = 0.004), and hypercholesterolemia (OR = 4.1, 95% CI = 1.2–14.1, p = 0.026), were also associated with serious NADEs. Seventeen cases (1.3%) with serious NADEs died among hospitalized cART-naive AIDS patients, and severe pneumonia (HR = 5.5, 95%CI = 1.9–15.9, p<0.001) and AIDS-defining cancers (HR = 3.8, 95%CI = 1.1–13.2, p = 0.038) were identified as risk factors associated with an increased hazard of mortality among these patients with serious NADEs. Serious NADEs also occurred in cART-naive AIDS patients in China with low prevalence. Our results reminded physicians that early screening of serious NADEs, timely intervention of their risk factors, management of severe AIDS-defining events, multi-disciplinary cooperation, and early initiation of cART were essential to reduce the burden of serious NADEs.

## Introduction

In spite of updated criteria of starting combination antiretroviral therapy (cART) and widespread coverage of cART in HIV-infected population in China [1], which reduced mortality and prolonged longevity among these patients, some patients were not administered cART for reasons such as unawareness of HIV infection, psychosocial pressure, or poor adherence [2]. Moreover, opportunistic infections (OIs) or AIDS-defining cancers (ADC) and serious non-AIDS-defining events (NADEs) were also major causes of morbidity and mortality [2, 3], which affected prognosis among these patients and increased the burden of healthcare in China.

Serious NADEs were gradually exacerbated by low-level chronic inflammation and immune activation in HIV-infected patients [4], which typically occurred in elderly general population and defined as immune senescence, which indicated that HIV-infected patients were experiencing premature ageing *in vivo* [5]. Serious NADEs included cardiovascular diseases, cerebrovascular diseases, liver cirrhosis, chronic kidney diseases, and non-AIDS-defining cancers (NADCs) [6, 7], which were typically senile diseases that occurred generally in elderly population. Serious NADEs were reported to be more common at a younger age in people with HIV than in the general population after initiating cART due to persistent immune senescence and residual inflammation [8]. Besides HIV-infected patients receiving cART, serious NADEs were also found to occur in HIV-infected patients prior to initiating cART due to immunodeficiency and viraemia [9].

The co-occurrence of serious NADEs and AIDS-defining events (ADEs), including OIs and ADCs, in cART-naive AIDS patients indicated difficulties of appropriate treatment, increased drug-drug interactions, and affected the prognosis among these patients [10]. The burden of serious NADEs among cART-naive AIDS patients was not clearly defined in China, and in this observational cohort, we retrospectively studied the burden, clinical characteristics, risk factors, and mortality of serious NADEs among admitted cART-naive AIDS patients. This helped increase awareness of physicians to timely screen serious NADEs and multi-disciplinary cooperation for diagnosis and treatment, as well as influence policy-makers to establish appropriate healthcare policy in the field of AIDS in China.

## Methods

### Ethics

This study complied with the principles of Declaration of Helsinki and was approved by Human Science Ethical committee of Beijing Ditan Hospital, Capital Medical University, the largest AIDS referral hospital in China. The Human Science Ethical committee authorized to waive informed consent based on observational nature of this study. The clinical information was abstracted from electronic medical records database and was studied anonymously in Ditan Hospital.

### Study participants

One-thousand three hundred and seventy cART-naive AIDS patients admitted in Beijing Ditan Hospital were identified between January 2009 and December 2018, who were unaware of HIV infection until opportunistic infections became the first indicator of their disease. These admitted patients presented unspecific symptoms, including fever with unknown origins, due to severe immunosuppression and etiologic diagnosis, and treatment was required. The patients were cART-naive individuals who were not exposed to any ARV regimens or ART history, had not initiated self-discontinuation, and were without discontinuation for other reasons. Eligible participants included those who were ≥18 years old and were cART-naive. Patients who were aged <18 years, cART-experienced, foreigners, and had incomplete clinical data were excluded. This implied that some admitted patients abandoned further etiologic diagnosis and treatment due to cost constraint [11], and we could not obtain their complete clinical data. The clinical characteristics, proportion, and risk factors of serious NADEs were evaluated in these AIDS patients, and risk factors of mortality were also further assessed in these patients with serious NADEs.

Among these cART-naive AIDS patients who were discharged and then readmitted due to immune reconstitution inflammatory syndrome, screening of opportunistic pathogens, biochemical tests, and imaging examination including CT scan were performed for etiologic diagnosis at first admission, including AIDS-defining events (ADEs) and non-AIDS-defining events (NADEs). Prior to initiating cART, we collected these clinical data at first admission but not those at readmission.

### Definitions and diagnosis

AIDS patients were defined as anti-HIV (+) patients with CD4 cell counts below 200 cells/ul and/or opportunistic infections or AIDS-defining cancers.

Serious NADEs were identified based on ICD-10 diagnostic codes and grouped in this study as: cardiovascular diseases, cerebrovascular diseases, liver cirrhosis, chronic kidney diseases and NADCs.

Cardiovascular diseases (CVD) were diagnosed based on medical history, which were further confirmed based on coronary angiography and echocardiography in patients with medical history, as well as signs and symptom of cardiovascular diseases.

Brain computerized tomography (CT) scan was conducted in admitted patients, in which patients with abnormal imaging finding or medical history, signs and symptom of cerebrovascular diseases would further receive screening based on magnetic resonance imaging (MRI) of brain or even cerebrovascular CT angiography.

Liver cirrhosis was classified as tissue fibrosis and the conversion of normal liver architecture into abnormal nodule due to long-term chronic hepatitis B or C infection with/without portal hypertension was based on imaging examination.

Chronic kidney diseases (CKD) was diagnosed based on estimated glomerular filtration rate (eGFR) <60 ml/min/1.73m^2^ and/or markers of renal damage for at least 3 months.

NADCs, excluding non-Hodgkin lymphoma, Kaposi’s sarcoma, and cervical cancer, were defined according to characteristic pathological diagnosis.

The diagnosis and treatment of opportunistic infections were carried out in Ditan Hospital in accordance with guidelines recommended by the National Institute of Health (NIH)of America [12]. The AIDS-defining cancers, including non-Hodgkin lymphoma, Kaposi’s sarcoma, and cervical cancer, were pathologically diagnosed and managed based on *HIVBOOK* published by Christian Hoffmann [13].

Dyslipidemia was diagnosed based on the following criteria: serum triglyceride (TG) ≥2.3mmol/L was defined as hypertriglyceridemia, total cholesterol (TC) ≥6.2mmol/L as hypercholesterolemia, low density lipoprotein (LDL) ≥3.12 mmol/L and high density lipoprotein (HDL) ≤1 mmol/L respectively, according to guideline on prevention and treatment of dyslipidemia in adults in China [14].

Severe pneumonia was diagnosed as severe infection in respiratory system by co-infections with bacteria, tuberculosis, virus and even fungi accompanied by compatible symptoms and signs [15].

The prognosis was documented either as death or survival among these patients when discharging from hospital.

### Data collection

AIDS patients were admitted due to various unspecific symptoms and signs, which may be caused by opportunistic infection, AIDS-defining cancers or even NADEs. Opportunistic pathogens screening, biochemical tests, and imaging examination including CT scan was performed routinely in all admitted patients.

The clinical information was abstracted from electronic medical records from January 2009 to December 2018. Demographic data included age and sex, and individual behavior data included cigarette smoking or alcoholic drinking history and HIV transmission routes. The diagnostic criteria of cigarette smoking history was reported smoking one or more cigarettes in the past day [16]. And the diagnostic criteria of alcohol drinking history in China was daily alcoholic intake was > 40 grams/day in men or > 20 grams/day in women for more than 5 years [17]. Laboratory data were collected including CD4 cell counts, CD4/CD8 ratio, lipid panel (TG, TC, LDL and HDL levels), hepatitis B antigen (HBsAg), anti-HCV antibody (anti-HCV), Treponema Pallidum Particle agglutination (TPPA), and Toludine Red Unheated Serum Test (TRUST). The clinical characteristics of cART-naive HIV/AIDS patients were recorded, including OIs or ADCs, serious NADEs, and other complications such as hypertension, diabetes mellitus type 2, syphilis and chronic hepatitis B or C.

### Statistical analysis

The statistical analysis was carried out using SPSS 24.0 (SPSS Institute, Chicago IL, USA). Categorical variables are presented as percentages and were compared using chi-squared tests, while continuous variables are expressed as median with interquartile ranges (IQR) and were compared using the student t tests for statistical continuous variables.

Univariate logistic regression models were first used to determine the association of the common risk factors in clinical work with having serious NADEs, and statistically significant predictors with P<0.1 were included in a subsequent multivariable logistic regression model. Model fit and potential collinearity were also evaluated in the variables.

Cox proportional hazard models were used to assess the risk factors of in-hospital mortality of serious NADEs among these patients with serious NADEs.

Alpha was set to 0.05 with two-side, and *p* value <0.05 was treated as statistically significant.

## Results

### Demographic and clinical features

Sixty-one of the 1370 cART-naive AIDS patients admitted in Beijing Ditan Hospital between January 2009 and December 2018 were excluded based on the recruitment criteria. The demographic and clinical features of 1309 included patients in this study are available in Table 1.

**Table 1:** Baseline demographics, clinical and laboratory features of serious non-AIDS-defining events among cART-naive HIV/AIDS patients.

Variables	Serious NADE-free	Serious NADEs	*p*	Serious NADEs							
				Age			*p*	CD4 cell			*p*
				<50 Years	50-65 Years	≥65 Years		>350 cells/ul	200-350cells/ul	≤200 cells/ul	
**Patients number (%)**	**1166(100)**	**143(100)**	**-**	**83(100)**	**43(100)**	**17(100)**	**-**	**12(100)**	**18(100)**	**113(100)**	**-**
**Age (yrs)** ^§^	**36(28-45)**	**46(37-58)**	**<0.001**	**39(32-44)**	**57(51-61)**	**69(67-73)**	**<0.001**	**47.5(41.5-65.5)**	**46(41.5-53)**	**45(35.5-58)**	**0.490**
**Age<50 years**	**995(85.3)**	**83(58.0)**	**<0.001**	**83(100)**	**-**	**-**	**-**	**7(58.3)**	**10(55.6)**	**66(58.4)**	0.642*
**50≤Age<65 years**	**156(13.4)**	**43(30.1)**		**-**	**43(100)**	**-**		**2(16.7)**	**7(38.9)**	**34(30.1)**	
**Age≥65 years**	**15(1.3)**	**17(11.9)**		**-**	**-**	**17(100)**		**3(25.0)**	**1(5.6)**	**13(11.5)**	
**Sex-Male (%)**	**1061(91.0)**	**127(88.8)**	**0.395**	**74(89.2)**	**36(83.7)**	**17(100)**	**0.080**^**▲**^	**9(75.0)**	**16(88.9)**	**102(90.3)**	0.154*
**Cigarette smoking history (%)**	**308(26.4)**	**55(38.5)**	**0.002**	**34(41.0)**	**17(39.5)**	**4(23.5)**	0.257*	**8(66.7)**	**6(33.3)**	**41(36.3)**	0.102*
**Alcohol drinking history (%)**	**257(22.0)**	**37(25.9)**	**0.300**	**21(25.3)**	**14(32.6)**	**2(11.8)**	0.600*	**5(41.7)**	**8(44.4)**	**24(21.2)**	**0.048**
**Transmission route**											
**Homosexual (%)**	**862(73.9)**	**107(74.8)**	0.589*	**61(73.4)**	**30(69.8)**	**16(94.1)**	**0.067**^**▲**^	**8(66.7)**	**14(77.8)**	**85(75.2)**	**0.231**^**▲**^
**Heterosexual (%)**	**198(17.0)**	**19(13.3)**		**10(12.0)**	**9(20.9)**	**0(0.0)**		**3(25.0)**	**4(22.2)**	**12(10.6)**	
**Blood transfusion (%)**	**90(7.7)**	**13(9.1)**		**8(9.6)**	**4(9.3)**	**1(5.9)**		**1(8.3)**	**0(0.0)**	**12(10.6)**	
**Intravenous drug (%)**	**16(1.4)**	**4(2.8)**		**4(4.8)**	**0(0.0)**	**0(0.0)**		**0(0.0)**	**0(0.0)**	**4(3.5)**	
**Laboratory Results**								**.**			
**CD4 (cells/ul)** ^§^	**40(13-189)**	**69(14-180)**	**0.957**	**72(15-183)**	**70(12-175)**	**65(29.5-230.5)**	**0.843**	**558(494.25-708)**	**281(246.5-301)**	**45(10.5-118.5)**	**<0.001**
**TC (mmol/L)**^§^	**3.25(2.73-3.94)**	**3.35(2.75-4.04)**	**0.482**	**3.28(2.42-3.91)**	**3.58(2.82-4.08)**	**3.21(2.74-4.21)**	**0.476**	**4.185(3.44-4.8025)**	**4.065(2.5375-4.425)**	**3.24(2.675-3.85)**	**0.005**
**TG (mmol/L)**^§^	**1.18(0.87-1.7325)**	**1.36(0.91-1.98)**	**0.053**	**1.30(0.84-1.95)**	**1.37(0.96-2.06)**	**1.36(0.80-1.94)**	**0.877**	**1.23(0.79-2.11)**	**1.64(0.86-2.07)**	**1.29(0.925-1.94)**	**0.395**
**HDL (mmol/L)**^§^	**0.66(0.51-0.8525)**	**0.68(0.52-0.81)**	**0.213**	**0.67(0.47-0.81)**	**0.74(0.60-0.84)**	**0.58(0.46-0.805)**	**0.287**	**0.765(0.72-0.975)**	**0.69(0.6-0.845)**	**0.65(0.475-0.805)**	**0.030**
**LDL (mmol/L)**^§^	**2.03(1.59-2.582)**	**2.1(1.59-2.51)**	**0.447**	**2.17(1.50-2.58)**	**2.05(1.59-2.51)**	**1.87(1.58-2.45)**	**0.872**	**2.645(1.785-3.0825)**	**2.16(1.3725-2.5725)**	**2.07(1.535-2.48)**	**0.136**
**CD4>350 cells/ul (%)**	**178(15.3)**	**12(8.4)**	**0.044**	**7(8.4)**	**2(4.7)**	**3(17.6)**	0.642*	**12(100)**	**-**	**-**	**-**
**200<CD4≤350cells/ul (%)**	**103(8.8)**	**18(12.6)**		**10(12.0)**	**7(16.3)**	**1(5.9)**		**-**	**18(100)**	**-**	
**CD4≤**20**0 cells/ul (%)**	**885(75.9)**	**113(79.0)**		**66(79.5)**	**34(79.1)**	**13(76.5)**		**-**	**-**	**113(100)**	
**CD4/CD8<1 (%)**	**1145(98.2)**	**140(97.9)**	1.000*	**80(96.4)**	**43(100)**	**17(100)**	**0.191**^**▲**^	**9(75.0)**	**18(100)**	**113(100)**	**<0.001**^**▲**^
**TC≥6.2mmol/L (%)**	**12(1.0)**	**4(2.8)**	0.158*	**3(3.6)**	**0(0.0)**	**1(5.9)**	**0.214**^**▲**^	**0(0.0)**	**2(11.1)**	**2(1.8)**	**0.147**^**▲**^
**TG≥2.3mmol/L (%)**	**143(12.3)**	**25(17.5)**	**0.078**	**15(18.1)**	**7(16.3)**	**3(17.6)**	0.885*	**3(25.0)**	**3(16.7)**	**19(16.8)**	0.553*
**HDL≤1mmol/L (%)**	**1017(87.2)**	**133(93.0)**	**0.046**	**78(94.0)**	**38(88.4)**	**17(100)**	**0.150**^**▲**^	**10(83.3)**	**16(88.9)**	**107(94.7)**	0.102*
**LDL≥3.12mmol/L (%)**	**104(8.9)**	**13(9.1)**	**0.946**	**6(7.2)**	**5(11.6)**	**2(11.8)**	0.406*	**3(25.0)**	**2(11.1)**	**8(7.1)**	0.048*
**AIDS-Defining Events**											
**CMV Infection (%)**	**112(9.6)**	**10(7.0)**	**0.310**	**6(7.2)**	**3(7.0)**	**1(5.9)**	0.857*	**0(0.0)**	**0(0.0)**	**10(8.8)**	**0.086**^**▲**^
**TB (%)**	**305(26.1)**	**33(23.1)**	**0.427**	**15(18.1)**	**13(30.2)**	**5(29.4)**	**0.247**	**0(0.0)**	**3(16.7)**	**30(26.5)**	**0.024**^**▲**^
**PCP (%)**	**76(6.5)**	**6(4.2)**	**0.279**	**4(4.8)**	**2(4.7)**	**0(0.0)**	**0.459**^**▲**^	**0(0.0)**	**0(0.0)**	**6(5.3)**	**0.235**^**▲**^
**Cryptococcosis (%)**	**68(5.8)**	**6(4.2)**	**0.424**	**4(4.8)**	**2(4.7)**	**0(0.0)**	**0.459**^**▲**^	**0(0.0)**	**0(0.0)**	**6(5.3)**	**0.235**^**▲**^
**Invasive fungal infection (%)**	**418(35.8)**	**40(28.0)**	**0.062**	**22(26.5)**	**12(27.9)**	**6(35.3)**	**0.763**	**0(0.0)**	**2(11.1)**	**38(33.6)**	**0.002**^**▲**^
**ADCs (%)**	**27(2.3)**	**7(4.9)**	**0.057**	**5(6.0)**	**2(4.7)**	**0(0.0)**	**0.382**^**▲**^	**0(0.0)**	**1(5.6)**	**6(5.3)**	**0.532**^**▲**^
**Severe pneumonia (%)**	**228(19.5)**	**37(25.9)**	**0.076**	**22(26.5)**	**8(18.6)**	**7(41.2)**	**0.194**	**0(0.0)**	**0(0.0)**	**37(32.7)**	**<0.001**^**▲**^
**Complications**											
**Diabetes (%)**	**26(2.2)**	**11(7.7)**	**<0.001**	**4(4.8)**	**4(9.3)**	**3(17.6)**	**0.069**	**1(8.3)**	**1(5.6)**	**9(8.0)**	0.906*
**Hypertension (%)**	**208(17.8)**	**60(42.0)**	**<0.001**	**24(28.9)**	**25(58.1)**	**11(64.7)**	**0.001**	**5(41.7)**	**9(50.0)**	**46(40.7)**	**0.759**
**Syphilis (%)**	**265(22.7)**	**33(23.1)**	**0.925**	**19(22.9)**	**11(25.6)**	**3(17.6)**	0.827*	**3(2.1)**	**2(11.1)**	**28(24.8)**	0.585*
**Chronic hepatitis B (%)**	**90(7.7)**	**16(11.2)**	**0.151**	**11(13.3)**	**4(9.3)**	**1(5.9)**	0.321*	**0(0.0)**	**1(5.6)**	**15(13.3)**	**0.135**^**▲**^
**Chronic hepatitis C (%)**	**46(4.0)**	**12(8.4)**	**0.015**	**10(12.0)**	**2(4.7)**	**0(0.0)**	**0.075**^**▲**^	**1(0.7)**	**2(11.1)**	**9(8.0)**	0.816*
**Serious NADEs**											
**NADCs (%)**	**-**	**37(25.9)**	**-**	**24(28.9)**	**10(23.3)**	**3(17.6)**	**0.284**	**6(4.2)**	**7(38.9)**	**24(21.2)**	**0.039**
**CVD (%)**	**-**	**26(18.2)**	**-**	**10(12.0)**	**9(20.9)**	**7(41.2)**	**0.015**	**3(2.1)**	**2(11.1)**	**21(18.6)**	0.898*
**Cerebrovascular Diseases (%)**	**-**	**49(34.3)**	**-**	**26(31.3)**	**17(39.5)**	**6(35.3)**	**0.652**	**2(1.4)**	**4(22.2)**	**43(38.1)**	0.067*
**Liver cirrhosis (%)**	**-**	**18(12.6)**	**-**	**12(14.5)**	**5(11.6)**	**1(5.9)**	0.332*	**1(0.7)**	**2(11.1)**	**15(13.3)**	<0.001*
**CKD (%)**	**-**	**28(19.6)**	**-**	**17(20.5)**	**7(16.3)**	**4(23.5)**	0.982*	**0(0.0)**	**4(22.2)**	**24(21.2)**	**<0.001**^**▲**^
**Death (%)**	**74(6.3)**	**17(11.9)**	**0.014**	**11(13.3)**	**2(4.7)**	**4(23.5)**	0.755*	**0(0.0)**	**5(27.8)**	**12(10.6)**	**0.014**^**▲**^

**Note**

*Continuous correction method

▲ Likelihood test

§ mean evaluation based on 25^th^, 75^th^ percentile; ADC: AIDS-defining cancer; ADE: AIDS-defining event; CKD: chronic kidney disease; CVD: cardiovascular disease; HDL: high density lipoprotein; LDL: low density lipoprotein; NADE: non-AIDS-defining event; TC: total cholesterol; TG: triglyceride; CMV:cytomegalovirus; TB:tuberculosis; PCP: Pneumocystis pneumonia; NADC: non-AIDS-defining cancer.

Of 1309 patients analyzed, median age was 38.2 years (18–78 years) and patients aged <50years, 50-65years and ≥65 years were 1078 cases (82.4%), 199 cases (15.2%) and 32 cases (2.4%), respectively. Cigarette smoking and alcohol drinking are presented among 363 cases**(**27.7%**)**and 294 cases (22.5%), respectively. Patients with CD4 ≤200cells/ul, 200–350, and >350cells/ul were 998 cases (76.2%), 121 cases (9.2%), and 190 cases (14.6%), respectively.

Besides serious NADEs, AIDS-defining events, including opportunistic infections (74.3%) and AIDS-defining cancers (2.7%), and other complications, including diabetes (2.8%), hypertension (20.5%), syphilis (22.7%), and chronic viral hepatitis B (8.1%) or C (4.5%), were diagnosed among these cART-naive AIDS patients.

The prognosis of those with and without serious NADEs was compared and we found that AIDS patients with serious NADEs presented the poorer prognosis compared to those without serious NADEs (p = 0.014) (Table 1).

## The burden of serious NADEs stratified by CD4 counts and age categories

Of 1309 admitted cART-naive AIDS patients in this study, 143 patients (10.9%) had at least one serious NADEs, which included cerebrovascular diseases in 49 patients (3.8%), non-AIDS-defining cancers in 37 patients (2.8%), CKD in 28 patients (2.1%), cardiovascular diseases in 26 patients (2.0%), and liver cirrhosis in 18 patients (1.4%).

**Fig 1:**
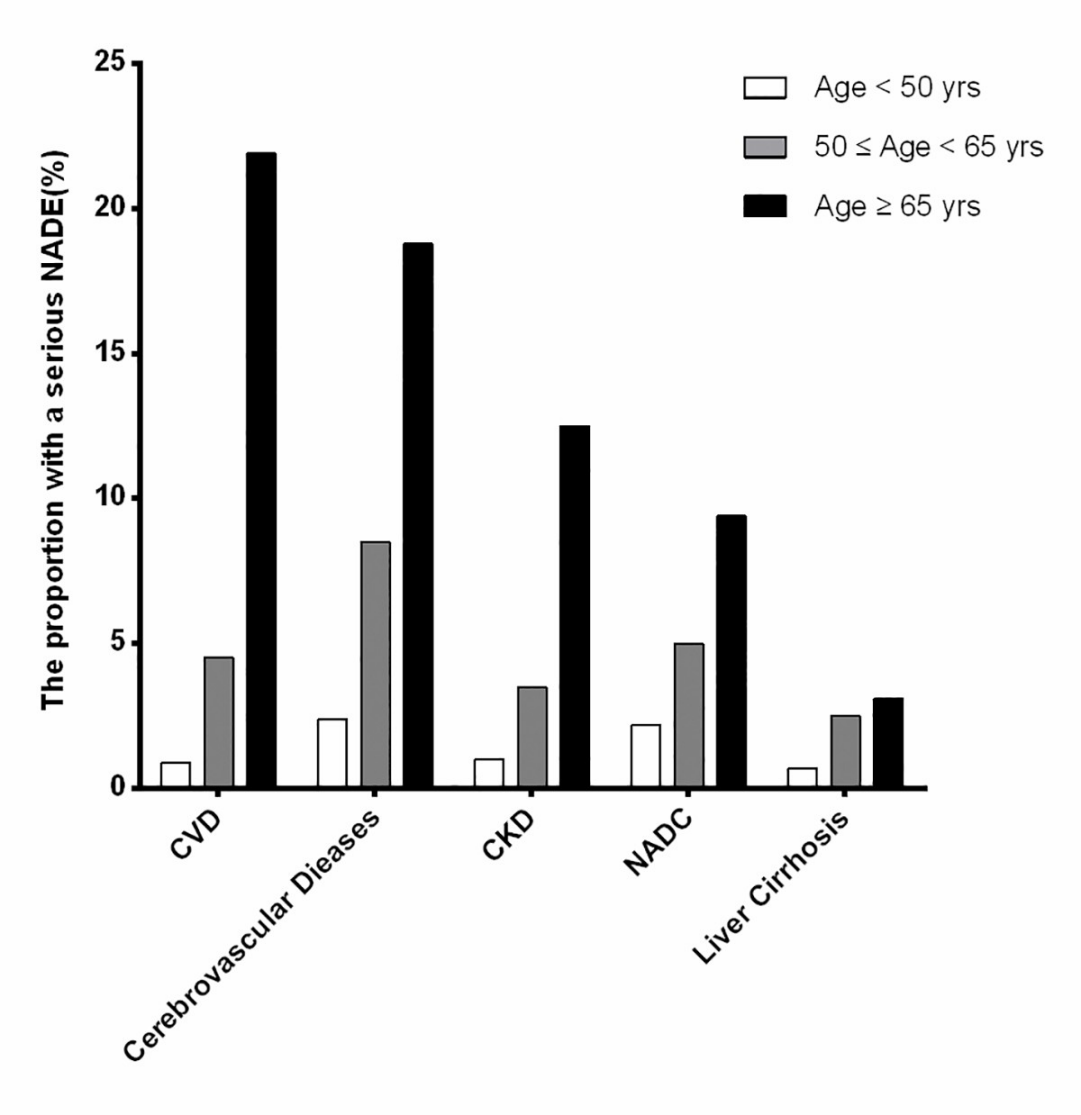
Proportion of cART-naive AIDS patients with serious NADEs stratified by age. The proportion is shown in three age strata (**<**50, 50–65 and **≥**65 years) in the order of their frequency. NADC: non-AIDS-defining cancer; NADE: non-AIDS-defining event; CKD: chronic kidney disease; CVD: cardiovascular disease.

The proportion with serious NADEs, stratified by age categories—<50 years, 50–65 years, and ≥65 years—increased with age, which indicated that serious NADEs were prone to occur in older cART-naive AIDS patients, and the burden of serious NADEs gradually increased in these older patients (see Fig 1).

**Fig 2:**
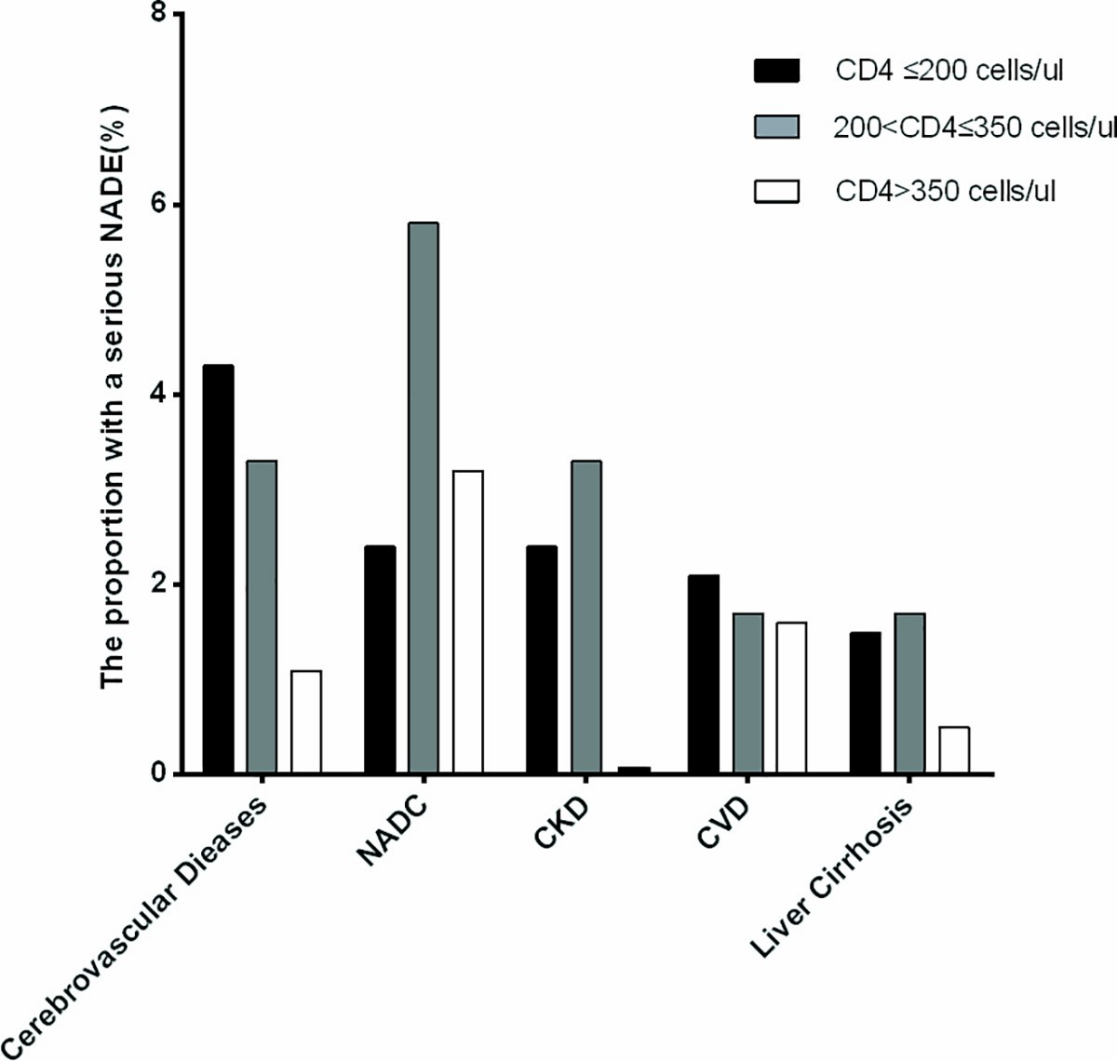
The proportion of cART-naive AIDS patients with serious NADEs stratified by CD4 levels. The proportion is shown in three CD4 cells counts strata (≤200, 200–350 and >350 cells/ul) in the order of their frequency. NADC: non-AIDS-defining cancer; NADE: non-AIDS-defining event; CKD: chronic kidney disease; CVD: cardiovascular disease.

The proportion with serious NADEs stratified by CD4 categories, CD4 counts ≤200cells/ul, 200–350, and >350cells/ul, indicated that these serious NADEs were distributed in different CD4 categories (see Fig 2).

### The burden of multiple serious NADEs stratified by CD4 counts and age categories

**Fig 3:**
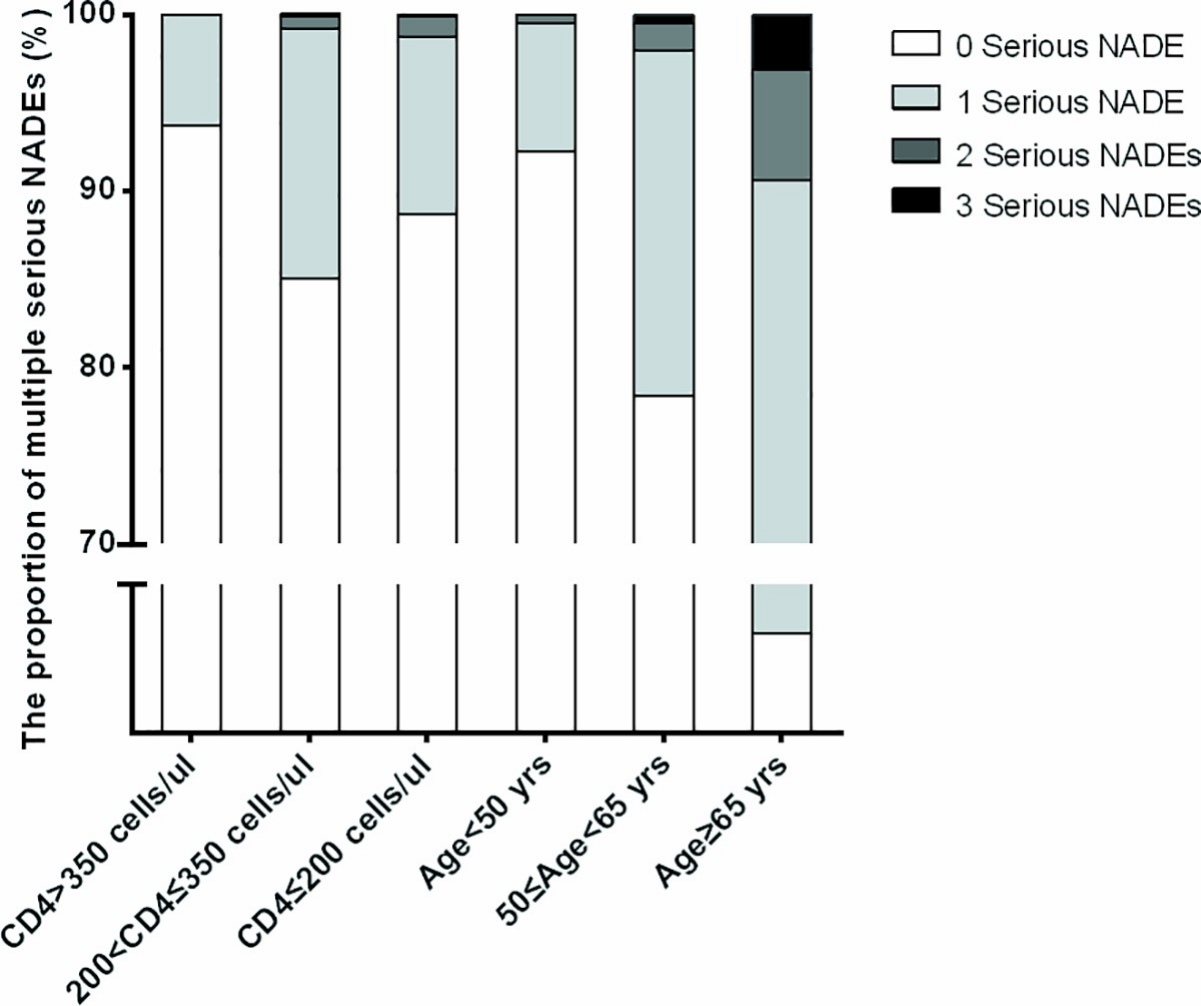
The proportion of multiple serious NADEs stratified by age and CD4 levels. NADE: non-AIDS-defining event.

We found that some cART-naive AIDS patients had one or more serious NADEs; the proportion of patients with multiple serious NADEs stratified by CD4 and age categories are described in Fig 3.

In patients with CD4 >350cells/ul, the proportion with 1 serious NADE was 6.3% (12/190 cases), but no one had 2 or 3 serious NADEs. The proportion with 1 and 2 serious NADEs was 14.1%(17/121 cases) and 0.8% (1/121 cases) in patients with CD4 between 200 and 350cells/ul, but no one had 3 serious NADEs, and that with 1, 2 and 3 serious NADEs was 10.1% (101/998 cases),1.0% (10/998 cases) and 0.2% (2/998 cases) respectively in patients with CD4 ≤200cells/ul.

In patients aged <50 years, the proportion with 1 and 2 serious NADEs was 7.2% (77/1078 cases) and 0.5% (5/1078 cases), respectively, but no one had 3 serious NADEs; in patients aged 50–65 years, the proportion with 1, 2 and 3 serious NADEs was 19.6% (39/199 cases), 1.5% (3/199 cases) and 0.5% (1/199 cases), respectively, and in patients aged ≥65 years, the proportion with 1, 2 and 3 serious NADEs was 43.7% (14/32), 6.3% (2/32 cases) and 3.1% (1/32 cases), respectively.

The prevalence in patients with CD4 ≤ 350 cels/ul and aged ≥ 50 years, CD4 ≤ 350 cels/ul and aged < 50 years, CD4 > 350 cels/ul and aged > 50 years, and with CD4 > 350 cels/ul and aged < 50 years was 27.4%, 8.3%, 1.7%, and 4.4%,m respectively (p<0.001), which indicated that the distribution of multiple serious NADEs was prone to co-occur in patients with CD4 ≤ 350 cels/ul and aged ≥ 50 years and increased complexity of diagnosis and management among cART-naive AIDS patients in China (S1 Table).

### Predictors of serious NADEs among cART-naive AIDS patients

In cART-naive AIDS patients, univariate logistic regression models were first used to determine the association of the common risk factors for having serious NADEs in clinical work and to identify statistically significant predictors (P<0.1). Age 50–65 years (OR = 3.3, 95%CI = 2.2–5.0, p<0.001), age ≥ 65 years (OR = 13.6, 95%CI = 6.6–28.2, p<0.001), cigarette smoking (OR = 1.7, 95%CI = 1.2–2.5, p = 0.003), CD4 within 200–350 cells/ul (OR = 2.6, 95%CI = 1.2–5.7, p = 0.014), CD4 ≤ 200 cells/ul (OR = 1.9, 95%CI = 1.0–3.5, p = 0.042), TC ≥ 6.2 mmol/L (OR = 2.8, 95% CI = 0.9–8.7, p = 0.081), TG ≥ 2.3 mmol/L (OR = 1.5, 95% CI = 1.0–2.4, p = 0.080), HDL ≤ 1.0 mmol/L (OR = 1.9, 95% CI = 1.0–3.8, p = 0.049), invasive fungal infections (OR = 0.7, 95% CI = 0.5–1.0, p = 0.063), AIDS-defining cancers (OR = 2.2, 95% CI = 0.9–5.1, p = 0.074), severe pneumonia (OR = 1.4, 95% CI = 1.0–5.1, p = 0.077), diabetes (OR = 3.7, 95% CI = 1.8–7.6, p<0.001), hypertension (OR = 2.2, 95% CI = 1.1–4.3, p<0.001) and chronic HCV infection (OR = 2.9, 95% CI = 1.5–5.6, p = 0.017), were included in a subsequent multivariate logistic regression model, in which age 50–65 years (OR = 2.6, 95%CI = 1.7–4.0, p<0.001), age ≥ 65 years (OR = 12.2, 95%CI = 5.6–26.5, p<0.001), cigarette smoking (OR = 1.9, 95%CI = 1.3–2.8, p = 0.002), CD4 within 200–350 cells/ul (OR = 3.0, 95%CI = 1.3–6.9, p = 0.009), CD4 ≤200 cells/ul (OR = 2.2, 95%CI = 1.1–4.4, p = 0.019), TC ≥ 6.2 mmol/L (OR = 4.1, 95% CI = 1.2–14.1, p = 0.026), hypertension (OR = 2.5, 95%CI = 1.7–3.7, p<0.001) and chronic HCV infection (OR = 2.8, 95%CI = 1.4–5.6, p = 0.004) were predictors of serious NADEs in cART-naive AIDS patients (Table 2).

**Table 2:** Risk factors for serious non-AIDS-defining events by logistic regression analysis among cART-naive AIDS patients.

Variables	Unadjusted		Adjusted	
	OR(95%CI)	*p*-value	OR(95%CI)	*p*-value
Age<50 years	1		1	
** 50≤Age<65 years**	**3.3(2.2–5.0)**	**<0.001**	**2.6(1.7–4.0)**	**<0.001**
** Age≥65 years**	**13.6(6.6–28.2)**	**<0.001**	**12.2(5.6–26.5)**	**<0.001**
** Sex-Male**	**0.8(0.5–1.4)**	**0.396**	**0.8(0.34–1.4)**	**0.426**
** Cigarette smoking history**	**1.7(1.2–2.5)**	**0.003**	**1.9(1.3–2.8)**	**0.002**
** Alcoholic drinking history**	**1.2(0.8–1.8)**	**0.301**	**0.9(0.6–1.5)**	**0.737**
** Laboratory Results**				
** CD4>350 cells/ul**	**1**		**1**	
** 200<CD4 cell≤350 cells/ul**	**2.6(1.2–5.7)**	**0.014**	**3.0(1.3–6.9)**	**0.009**
** CD4 cell≤200 cells/ul**	**1.9(1.0–3.5)**	**0.042**	**2.2(1.1–4.4)**	**0.019**
** CD4/CD8<1**	**0.9(0.3–2.9)**	**0.803**	**0.7(0.2–2.8)**	**0.572**
** TC≥6.2 mmol/L**	**2.8(0.9–8.7)**	**0.081**	**4.1(1.2–14.1)**	**0.026**
** TG≥2.3 mmol/L**	**1.5(1.0–2.4)**	**0.080**	**1.3(0.8–2.2)**	**0.179**
** HDL≤1 mmol/L**	**1.9(1.0–3.8)**	**0.049**	**1.6(0.8–3.3)**	**0.125**
** LDL≥3.12 mmol/L**	**1.0(0.6–1.9)**	**0.946**	**0.7(0.3–1.5)**	**0.337**
** AIDS-defining events**				
** CMV Infection**	**0.7(0.4–1.4)**	**0.313**	**1.0(0.5–1.9)**	**0.506**
** TB**	**0.8(0.6–1.3)**	**0.427**	**0.8(0.5–1.2)**	**0.304**
** PCP**	**0.6(0.3–1.5)**	**0.284**	**0.7(0.3–1.7)**	**0.346**
** Cryptococcus Infection**	**0.7(0.3–1.7)**	**0.426**	**0.7(0.3–1.7)**	**0.412**
** Invasive Fungal Infection**	**0.7(0.5–1.0)**	**0.063**	**0.6(0.4–1.0)**	**0.057**
** AIDS-defining cancers**	**2.2(0.9–5.1)**	**0.074**	**1.9(0.7–4.7)**	**0.116**
** Severe pneumonia**	**1.4(1.0–5.1)**	**0.077**	**1.3(0.8–2.1)**	**0.634**
** Complications**				
** Diabetes**	**3.7(1.8–7.6)**	**<0.001**	**1.6(0.7–3.9)**	**0.157**
** Hypertension**	**2.2(1.1–4.3)**	**<0.001**	**2.5(1.7–3.7)**	**<0.001**
** Syphilis**	**1.0(0.7–1.5)**	**0.925**	**1.1(0.7–1.8)**	**0.653**
** Chronic hepatitis B**	**1.5(0.9–2.6)**	**0.154**	**1.6(0.9–3.0)**	**0.064**
** Chronic hepatitis C**	**2.9(1.5–5.6)**	**0.017**	**2.8(1.4–5.6)**	**0.004**

**Note:** ADC: AIDS-defining cancer; HDL: high density lipoprotein; LDL: low density lipoprotein; TC: total cholesterol; TG: triglyceride; CMV: cytomegalovirus; TB: tuberculosis; PCP: Pneumocystis Pneumonia; OR: Odds Ratio.

We also assessed potential collinearity in the variables in multivariate logistic regression model, and we found that the Nagelkerke R^2^ value was 0.164. The variance inflation factor (VIF) value was calculated based on formula: VIF = 1/(1-R^2^), and result was 1.2; this is less than 5, which indicates that we can ignore VIF to influence parameters’ fit in the model. More importantly, although potential collinearity resulted in increased coefficient’s estimated variance, the predictive ability of the model did not decrease, which indicated the overfitting can be excluded in this model.

### Mortality and survival analysis among cART-naive AIDS patients with serious NADEs

Overall, 17 patients (1.3%) with serious NADEs died during hospitalization among the cART-naive AIDS patients, including CKD in 9 patients (0.7%), cerebrovascular diseases in 5 patients (0.3%), CVD in 3 patients (0.2%) and NADC in 1 patient (0.1%), in which included CVD and CKD co-occurred in 1 patient.

There were 17 deaths while there were 23 parameters in the multivariable model, which demonstrated overfitting, we chose Univariable COX proportional hazard regression analysis, which demonstrated that severe pneumonia (HR = 5.5, 95%CI = 1.9–15.9, p<0.001) and AIDS-defining cancers (HR = 3.8, 95%CI = 1.1–13.2, p = 0.038) as risk factors were associated with an increased hazard of mortality. Due to the most common and severe complication in AIDS patients in China [11], we elucidated the effect of severe pneumonia, Kaplan-Meier survival curves were plotted, stratified with or without severe pneumonia, log-rank testing indicated significant difference between 2 groups, which indicated that severe pneumonia increased mortality among cART-naive AIDS patients with serious NADEs (S2 Table & S1 Fig).

## Discussion

Despite availability of National Free Antiretroviral Treatment Programs (NFATP) in China [18, 19], some HIV-infected patients were not aware of HIV infection until opportunistic infections became the first indicator of their disease [2]. Previous studies reported that NADEs occurred among AIDS patients on ART, while studies on NADEs among cART-naive or cART-experienced AIDS patients in China were limited. In this study, except AIDS-defining events (including opportunistic infections and AIDS-defining cancers), we attempted to elucidate the burden, risk factors, and prognosis of serious Non-AIDS-defining events (NADEs) among cART-naive AIDS patients.

Besides AIDS-defining events, NADEs also occurred among cART-naive AIDS patients due to persistent chronic inflammation, immunosuppression, and viraemia [9], which included dyslipidemia, diabetes, hypertension, osteoporosis, chronic kidney diseases, cardio-cerebrovascular diseases, liver cirrhosis, and non-AIDS-defining cancers [20]. Of these, chronic kidney diseases, cardio-cerebrovascular diseases, liver cirrhosis, and non-AIDS-defining cancers were serious NADEs [6, 7] and increased the complexity of disease and affected the prognosis among cART-naive AIDS patients.

It was reported that [21], compared with general population, non-AIDS related mortality was 8.1 times higher in HIV-infected patients, which indicated that excess mortality was maintained in HIV-infected patients and indicated importance of screening for NADEs.

This is the first study to evaluate the burden, risk factors, and prognosis of serious NADEs in cART-naive AIDS patients, using electronic medical records database in China. We found that serious NADEs were also another major complications in AIDS patients prior to receiving cART besides AIDS-defining events, and serious NADEs could occur among cART-naive AIDS patients with older age or lower CD4 categories. This finding indicated that cART-naive AIDS patients, especially older patients, experienced serious NADEs earlier. Moreover, this finding demonstrates the importance of earlier initiation of cART, which inhibited viremia, reduced inflammatory cytokines *in vivo*, and helped increase awareness of healthcare workers to establish appropriate diagnostic and treatment strategies for these patients.

Hsue *et al*. [22] reported that increased atherosclerosis can occur in cART-naive AIDS patients due to persistent chronic inflammation, which accounted for earlier burden of cardiovascular and cerebrovascular diseases among cART-naive AIDS patients. Our results in this study also indicated that, despite low prevalence, the proportion of cardiovascular and cerebrovascular diseases gradually increased with the older age and decline of CD4 levels, which indicated that cardiovascular and cerebrovascular diseases should be found earlier among cART-naive AIDS patients and underestimation should be alerted.

It was reported that [23] the burden of NADCs increased with advancing age, especially in HIV-infected patients aged ≥50 years, and different NADCs occurred in different categories of CD4 levels. Our results indicate that NADCs also occurred in cART-naive AIDS patients with low prevalence, the proportion of NADCs gradually increased with age, and NADCs were distributed in different categories of CD4 levels, which indicated the importance of earlier screening NADCs among these patients in China.

Some studies reported that CKD occurred in 3.5%-48.5% of HIV-infected population [24], and older age and lower CD4 levels were its risk factors. Our results indicated that the burden of CKD was 2.1% among cART-naive AIDS patients in China, which increased with age and mainly occurred in patients with CD4 ≤ 350 cells/ul. This indicated that, despite low prevalence, timely detection of eGFR and earlier finding of CKD were beneficial to establish appropriate antiretroviral regimens for these patients.

HIV infection was a risk factor for liver fibrosis [25] and accelerated the progression of liver fibrosis among HIV/HBV or HIV/HCV co-infected patients [26]. Our results indicated that the burden of liver cirrhosis was 1.4% among cART-naive AIDS patients, and was distributed in older age and different categories of CD4 levels. This indicated, regardless of CD4 levels, earlier ultrasound screening to find occult liver fibrosis was necessary, especially among HIV/HBV or HIV/HCV co-infected patients.

The results in our study revealed that serious NADEs could occur in cART-naive AIDS patients with different CD4 levels, especially with CD4 ≤ 350 cells/ul, which was consistent with results previously found in industrialize countries [26]. Salter *et al*. demonstrated that untreated HIV infection was related to an increasing number of non-AIDS-defining events, and immunosuppression and uncontrolled HIV viremia further enhanced the effect [27]. One possible biological mechanism may be that untreated HIV infection itself was related to reduced CD4 cell counts and increased levels of virally mediated inflammatory biomarkers [28], coagulation imbalance [29], and endothelial cell dysfunction [30], which were all predictors for contributing to development of NADEs. The results in this study also revealed that serious NADEs could occur in cART-naive AIDS patients with CD4 > 350 cells/ul, which demonstrated that serious NADEs could occur in cART-naive patients with higher CD4 levels and earlier screening was necessary among these patients.

In general population, serious NADEs were senescence-associated diseases caused by chronic inflammations in elderly patients in vivo [20], while in HIV-infected population, HIV accelerated ageing process and affected prognosis of AIDS patients and came to the attention of clinicians [20, 31]. In this study, we also demonstrated that serious NADEs occurred in cART-naive AIDS patients of different ages, especially those aged ≥ 50 years, and further investigation indicated multiple serious NADEs also distributed among different age categories. The possible explanation was normal senescence process was accelerated by pre-matured ageing caused by persistent inflammatory factors induced by uncontrolled HIV infection [31].

In this study, all patients were HIV-infected patients with end stage of AIDS, who presented unspecific symptoms; prior to initiating cART, diagnosis and treatment of AIDS-defining events (ADEs), including opportunistic infections and AIDS-defining cancers, were first considered. Besides ADEs, we found serious NADEs could occur different CD4 levels and age and increased mortality. We suggest that, in addition to screening for opportunistic pathogens, earlier screening and treatment of serious NADEs among cART-naive AIDS patients after admission and/or prior to initiating cART, but not after taking cART, was beneficial for improving quality of lives of patients.

It was reported that, besides liver fibrosis and cirrhosis, extra-hepatic comorbidities including malignancies, cardiovascular diseases, CKD, and neurological diseases were well established in HCV-infected population [32]. Chronic viral infection, including HIV and HCV, induced to generate systemic chronic inflammation, which was more prone to cause serious noncirrhotic liver diseases (NCDs) via some mechanisms including oxidative stress and mitochondrial dysfunction [33]. In this study, we found that chronic HCV infection as a traditional risk factor was associated with serious NCDs among cART-naive AIDS patients, which reminded physicians of screening hepatitis C infection in HIV-infected population and of timely initiating treatment of direct-acting antiviral agents (DAA) in HIV/HCV co-infected patients.

Hypertension was reported as a common complication in HIV-infected patients, and we found that the prevalence of hypertension was 20.5% in our cohort, which was similar with results reported in developed countries [34]. It was well established that hypertension increased the risk of CVD, cerebrovascular events, chronic renal damage, and even liver fibrosis in general population [35], and we found that hypertension as a traditional risk factor was associated with serious NADEs in cART-naive AIDS patients, which indicated that monitoring and controlling blood pressure under normal scope helped reduce the risk of serious NADEs.

It was reported that, in HIV-infected patients, lower serum TC and mild increased TG levels were found prior to cART initiated [36, 37], and dyslipidemia as a traditional risk factor was associated with atherosclerosis [38]. In this study, we also found that, despite low prevalence, hypercholesterolemia was a risk factor was associated with serious NADEs in cART-naive AIDS patients, which highlighted physicians that it was important to screen and control hyperlipidemia in these patients.

It was reported that lower CD4/CD8 ratio [39] and chronic viral reactivation such as cytomegalovirus [40] as risk factors were associated with non-AIDS-defining diseases, but our results were inconsistent with these results in this study, indicating that these variables were considered to be treated as competing factors introducing confounders in assessment of risk factors of serious NADEs, and other risk factors including age and CD4 stratification became main predictors in multivariable logistic regression model.

It was reported that people living with HIV receiving cART had greater burden of NADEs, who tended to have one or more NADEs [41]. In this study, we also demonstrated that, in cART-naive AIDS patients, one or more serious NADEs were prone to co-occur among some patients with age ≥ 50 years and/or CD4 ≤ 350 cells/ul. The possible mechanisms, including uncontrolled chronic inflammation, immunodeficiency and viraemia, accelerated pre-matured senescence process [5, 31], resulting in multiple serious NADEs, which increased complexity of diseases, influenced the prognosis of patients and emphasized the importance of systemic screening and multi-disciplinary cooperation for diagnosis and therapy in these patients.

Some literature reported that AIDS-defining illnesses as a significant drivers were associated with pathogenesis of NADEs-related death in spite of unmeasured confounders due to some fatal AIDS-defining illnesses, including Pneumocystis pneumonia, and treatment and prophylaxis of AIDS-defining illnesses and further initiation of cART reduced the mortality of NADEs [42]. In this study, 17 cART-naive AIDS patients with serious NADEs died, including CKD in 9 patients, cerebrovascular diseases in 5 patients, CVD in 3 patients, and NADC in 1 patient, in which included CVD and CKD co-occurred in 1 patient. In this Cox proportional hazard models, our results indicated that severe pneumonia and AIDS-defining cancers were associated with mortality in cART-naive AIDS patients with serious NADEs. Except 4 dead cases due to severe pneumonia, cause of death in other 13 cases were serious NADEs but not severe pneumonia and AIDS-defining cancers, which may be due to deterioration of serious NADEs caused by acute inflammation in severe pneumonia and cancers. Our results were consistent with those reported in developed countries [42] and indicated that severe AIDS-defining events could drive NADEs-related death among cART-naive AIDS patients with serious NADEs. Moreover, they suggest that timely treatment and earlier initiating cART are beneficial for improving prognosis among these patients.

The results in this study revealed burden, risk factors, and prognosis of serious NADEs in cART-naive AIDS patients, in which OIs or ADCs were also common complications in these patients, especially in those with CD4 less than 200 cells/ul, which undoubtedly added complexity to diagnosis and treatment among these patients. Although co-medications were out range of this study, it was not surprising that co-medications [43], including management of OIs or ADCs and serious NADEs, followed by cART, were necessary in these patients, and it was important to notice the adverse effects as result of co-medications. The patients receiving multiple co-medications including management of OIs or ADCs, serious NADEs and even initiating cART increased potential risk of drug-drug interaction among them and resulted in poor adherence, which reminded healthcare workers in China that it was necessary to closely monitor potential adverse effects and drug-drug interaction among these patients.

The study about the burden, risk factors, and prognosis of serious NADEs in cART-naive AIDS patients has some strengths. First, serious NADEs were previously reported to occur in cART-experienced AIDS patients [6, 7], whereas in this study, we found that serious NADEs also occurred in cART-naive patients in China, which helped remind healthcare workers of the importance of screening serious NADEs besides AIDS-defining events and further reduce mortality in these patients. Second, the clinical data were abstracted from an observational cohort over a 10-year period, which indicated reliability of the conclusions. Third, we previously reported that AIDS patients in Ditan Hospital came from different geographic regions in China [2], which indicated that burden, risk factors, and mortality of serious NADEs found in this study showed its representativeness and universality in cART-naive AIDS patients in China.

The study had some limitations: First, potential bias inherently existed in retrospective and observational study. Second, only 1309 cART-naive AIDS patients were included in this retrospective and observational cohort, which indicated that it was not a meta-cohort study and the results should be further validated with the increased number of patients. Third, a further limitation of the study was that it was a hospital-based rather than population-based cohort. Patients admitted to hospital were more likely to have a serious NADE, and so the true prevalence of serious NADEs among cART-naïve patients in China may be lower. Despite the limitations, this study provided insight about the burden, risk factors and mortality of serious NADEs in cART-naive AIDS patients in China.

In summary, serious NADEs, including cardiovascular diseases, cerebrovascular diseases, liver cirrhosis, chronic kidney diseases, and NADCs, also occurred in cART-naive AIDS patients in China in addition to AIDS-defining diseases, despite low prevalence. We found these serious NADEs distributed in different age and CD4 levels, which indicated earlier screening of serious NADEs, regardless of age and CD4 levels, was necessary. Other traditional risk factors, including cigarette smoking, hypertension, chronic HCV infection and hypercholesterolemia, were also associated with serious NADEs, and severe AIDS-defining events as a significant drivers were associated with serious NADEs-related death, which indicated earlier intervention of these risk factors helped reduce the occurrence of serious NADEs and its related death. Our results will remind physicians that earlier screening and intervention of serious NADEs are necessary, and multi-disciplinary cooperation and earlier initiation of cART is essential to reduce the burden of serious NADEs.

## Supporting information

S1 Fig Survival curve for HIV-infected patients with or without severe pneumonia. To clarify the effects of severe pneumonia on survival, Kaplan-Meier survival curves were plotted for study subjects stratified by variable with/without severe pneumonia, which indicated that there was significant difference between the two groups (p<0.001).
(TIF)

S1 Table The distribution of serious NADEs based on age and CD4 levels among cART-naive AIDS patients. (DOCX)

S2 Table Risk factors for mortality analyzed with Cox proportional hazard regression among. (DOCX)

S1 Data (XLS)
