# Supplementary figures and images for: The burden of serious non-AIDS-defining events among admitted cART-naive AIDS patients in China: An observational cohort study
Source: PLoS One. 2020 Dec 22;15(12):e0243773. doi: 10.1371/journal.pone.0243773 (PMC7755215; doi:10.1371/journal.pone.0243773)

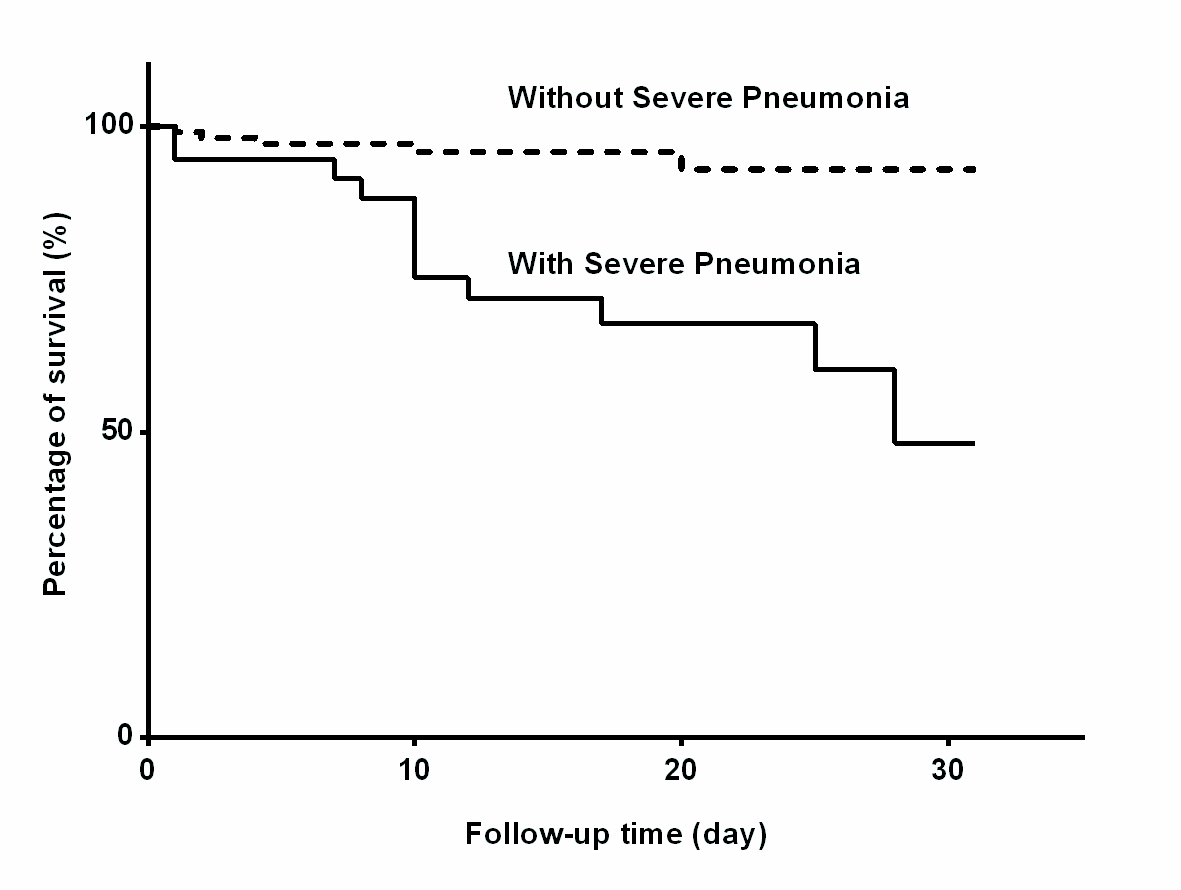

Supplement: S1 Fig — To clarify the effects of severe pneumonia on survival, Kaplan-Meier survival curves were plotted for study subjects stratified by variable with/without severe pneumonia, which indicated that there was significant difference between the two groups (p<0.001). (TIF) [file pone.0243773.s001.tif]
