# Supplementary material for: The burden of serious non-AIDS-defining events among admitted cART-naive AIDS patients in China: An observational cohort study
Source: PLoS One. 2020 Dec 22;15(12):e0243773. doi: 10.1371/journal.pone.0243773 (PMC7755215; doi:10.1371/journal.pone.0243773)
Supplement: S1 Table — (DOCX) [file pone.0243773.s002.docx]

**S1 Table：The distribution of serious NADEs based on age and CD4 levels among cART-naive AIDS patients.**

|  | | | | | |
| --- | --- | --- | --- | --- | --- |
|  | **Age≥50 yrs**  **&CD4≤350 cells/ul** | **Age<50 yrs**  **&CD4≤350 cells/ul** | **Age≥50 yrs**  **&CD4>350 cells/ul** | **Age<50 yrs**  **&CD4>350 cells/ul** | ***p*** |
| **Serious NADEs** | **55** | **76** | **5** | **7** | **<0.001** |
| **Total cases** | **201** | **918** | **30** | **160** |  |
| **Prevalence** | **27.4%** | **8.3%** | **1.7%** | **4.4%** |  |

**Note**: NADE: non-AIDS-defining event.
