# Supplementary material for: The burden of serious non-AIDS-defining events among admitted cART-naive AIDS patients in China: An observational cohort study
Source: PLoS One. 2020 Dec 22;15(12):e0243773. doi: 10.1371/journal.pone.0243773 (PMC7755215; doi:10.1371/journal.pone.0243773)
Supplement: S2 Table — (DOCX) [file pone.0243773.s003.docx]

**S2 Table:** Risk factors for mortality analyzed with Cox proportional hazard regression among.

|  | | | |
| --- | --- | --- | --- |
| **Variables** | **Death (%)** | **Unadjusted**  **HR (95%CI)** | **p-value** |
| **Age<50 years** | **11(7.7)** | **1** | **-** |
| **50≤Age<65 years** | **2(1.4)** | **0.3(0.1-1.5)** | **0.153** |
| **Age≥65 years** | **4(2.8)** | **1.6(0.5-5.1)** | **0.417** |
| **Sex-Male** | **16(11.2)** | **1.8(0.2-13.3)** | **0.584** |
| **Cigarette smoking history** | **4(2.8)** | **0.6(0.2-1.9)** | **0.384** |
| **Alcoholic drinking history** | **3(2.1)** | **0.7(0.2-2.3)** | **0.519** |
| **Laboratory Results** |  |  |  |
| **CD4>350 cells/ul** | **0(0.0)** | **-** | **-** |
| **200<CD4 cell≤350 cells/ul** | **0(0.0)** | **-** | **-** |
| **CD4 cell≤200 cells/ul** | **17(11.9)** | **-** | **-** |
| **TG≥2.3 mmol/L** | **3(2.1)** | **1.1(0.3-3.9)** | **0.847** |
| **HDL≤1 mmol/L** | **16(11.2)** | **1.0(0.1-7.8)** | **0.976** |
| **AIDS-defining events** |  |  |  |
| **CMV Infection** | **0(0.0)** | **-** | **-** |
| **TB** | **1(0.7)** | **0.2(0.2-1.3)** | **0.086** |
| **PCP** | **0(0.0)** | **-** | **-** |
| **Cryptococcus Infection** | **1(0.7)** | **1.5(0.2-11.7)** | **0.672** |
| **Invasive Fungal Infection** | **7(4.9)** | **1.2(0.4-3.2)** | **0.713** |
| **Severe Pneumonia** | **12(8.4)** | **5.5(1.9-15.9)** | **<0.001** |
| **AIDS-defining cancers** | **3(2.1)** | **3.8(1.1-13.2)** | **0.038** |
| **Complications** |  |  |  |
| **Diabetes** | **2(1.4)** | **1.6(0.4-6.8)** | **0.554** |
| **Hypertension** | **9(6.3)** | **1.4(0.6-3.7)** | **0.456** |
| **Syphilis** | **3(2.1)** | **0.7(0.2-2.4)** | **0.549** |
| **Chronic hepatitis B** | **2(1.4)** | **0.9(0.2-4.1)** | **0.919** |
| **Chronic hepatitis C** | **0(0.0)** | **-** | **-** |

**Note:** There were 17 deaths reported among those with serious NADE resulting in insufficient power to explore independent predictors of mortality in multivariable analysis.

ADC: AIDS-defining cancer; CMV: cytomegalovirus; TB: tuberculosis; PCP: Pneumocystis Pneumonia; HR: Hazard Ratio.
